# Reduced bio-efficacy of permethrin EC impregnated bednets against an *Anopheles gambiae *strain with oxidase-based pyrethroid tolerance

**DOI:** 10.1186/1475-2875-3-46

**Published:** 2004-11-29

**Authors:** Josiane Etang, Fabrice Chandre, Pierre Guillet, Lucien Manga

**Affiliations:** 1 Institute of Medical Research and Studies of Medicinal Plants (IMPM), Ministry of Scientific Research and Technique, P.O. Box 6163, Yaoundé, Cameroon; 2 Organisation de Coordination pour la lutte contre les Endémies en Afrique Centrale (OCEAC), Tel. +237-223-22-32, Fax. +237-223-00-61 BP. 288, Yaoundé, Cameroun; 3 Centre de Recherches Entomologiques de Cotonou (CREC), 06 B. P. 2064, Cotonou, Bénin; 4 World Health Organization, Head office, Geneva, Switzerland; 5 World Health Organization, Regional Office for Africa, P.O. Box BE 773, Harare, Zimbabwe

## Abstract

**Background:**

Insecticide-treated nets (ITNs) are an integral component of malaria control programmes in Africa. How much pyrethroid resistance in malaria vectors will impact on the efficacy of ITNs is controversial. The purpose of this study was to evaluate knockdown and killing effects of ITNs on a metabolic-based resistant or tolerant malaria vector strain.

**Methods:**

Bio-efficacy of 500 mg/m^2 ^permethrin EC treated bednets was assessed on the OCEAC laboratory (OC-Lab) strain of *Anopheles gambiae *s.s.. This strain is resistant to DDT and tolerant to pyrethroids, with elevated mixed function oxidases. The Kisumu reference susceptible strain of *A. gambiae s.s*. was used as control. Nets were impregnated in February 1998 and used by households of the Ebogo village. Then they were collected monthly over six months for Bio-assays (WHO cone test). Knockdown and mortality rates were compared between the OC-Lab and the Kisumu strains, by means of the Mantel-Haenszel chi-square test.

**Results:**

During the whole trial, permethrin EC knockdown rates were impressive (mostly higher than 97%). No significant difference was observed between the two strains. However, the mortality rates were significantly decreased in the OC-Lab strain (40–80%) compared with that of the Kisumu strain (75–100%). The decrease of killing effect on the OC-Lab strain was attributed to permethrin EC tolerance, due to the high oxidase metabolic activity.

**Conclusion:**

These data suggested an impact of pyrethroid tolerance on the residual activity of ITNs. More attention should be given to early detection of resistance using biochemical or molecular assays for better resistance management.

## Background

Malaria is the most important vector-borne disease in Africa. It is estimated that 80 to 90% of the 300 million annual cases and one million deaths occur on this continent [1]. The sharp rise of its incidence in the past decades resulted in dramatic economic consequences for African countries [2]. The global strategy adopted by the World Health Organization (WHO) in 1992 recommended an integrated management of the disease, including selective vector control [3]. Selective vector control is defined as: application of site-specific targeted use of different and cost-effective vector control methods alone or in combination to reduce human-vector contact.

Insecticide-treated nets (ITNs) are one of the main vector control tools against malaria. They are as effective as indoor residual spraying (IRS) [4] and strongly advocated for malaria prevention [5,6]. Implementation does not systematically require vector control services that no longer exist in many countries. At this time, insecticides belonging to the pyrethroid family are the only compounds available for the impregnation of materials. They strike mosquitoes with knockdown and killing effects at dosages far below the threshold of mammalian toxicity [7].

However, the emergence of pyrethroid resistance in the *Anopheles gambiae *complex and the *Anopheles funestus *group, the most important malaria vectors in Africa, is a threat to the effectiveness of ITNs [8-11]. This resistance is based on several mechanisms that could segregate according to their operational impact on vector biology and control. Some modifications of insecticide effects associated with reduced sensitivity of the sodium ion channel along nerve axons due to *kdr *mutation have been reported in *A. gambiae *s.s. from West and East Africa [12,13]. In addition, there is strong evidence for metabolic-based resistance mechanisms in African malaria vectors [14,15]. Three major enzyme families (esterases, glutathione S-transferases and cytochrome P_450 _oxidases) are involved in insect detoxification. Elevation of their activity usually results in resistance to insecticides such as pyrethroids. In Cameroon, elevated esterase, oxidase or glutathione S-transferase activities were reported as the main resistance mechanisms in many populations of the *A. gambiae *complex [16].

Malaria vector resistance to pyrethroids has been clearly demonstrated in Africa. However, its operational implication in terms of reducing efficacy of ITNs, especially in the case of metabolic-based resistance is not well documented. The aim of this study was to assess the knockdown and killing effects of ITNs on a metabolic-based pyrethroid resistance or tolerance strain of malaria vector. This study reports on the decrease of ITN's killing effect against a laboratory strain of *A. gambiae *s.s with a likely oxidase-based pyrethroid tolerance.

## Methods

The study was undertaken in the entomology laboratory of the Organisation de Coordination pour la lutte contre les Endémies en Afrique Centrale (OCEAC), in Yaoundé (Cameroon).

### Bednets impregnation and sampling

Bednets were made of white multifilament polyester fabric (75 denier; 156 meshes, 12 × 13 holes/inch^2^) manufactured by SiamDutch Mosquito Netting Co. Ltd. (Bangkok, Thailand). Two sizes of bednets were used : X-family (16.3 m^2^) and Family (13.13 m^2^). Both were strengthened on the lower part by a 20 cm sheeting border (made of more polyester filaments) to prevent tearing while being tucked in. They were impregnated with the target dosage of 500 mg/m^2 ^permethrin EC and hung in households of the Ebogo village, for use during the period of March-September, 1998.

Ebogo-village (3°20 N, 11°20 E) is about 65 km far from Yaoundé (the capital city of Cameroon), in the equatorial forest. *Anopheles moucheti *is the main malaria vector there, with 307 infected bites/man/year [17]. This village was chosen for the implementation of ITNs because the people there were used to bednets, since a deltamethrin SC trial was conducted there in 1994.

A total of 50 permethrin EC impregnated bednets were distributed in the village, in addition to about 30 old nets that were retreated by the study team. All the new nets were identified by a code number. Immediately after impregnation, two nets were randomly chosen and brought to the laboratory. Then, two others were collected each month from the Ebogo households and systematically replaced by unused ones. Replacement nets and old ones were properly identified so that they could not later be collected from the field and used for bio-assays. People were asked not to wash their bednets during the trial.

### Laboratory procedure

#### Netting section

In the laboratory, netting portions were isolated from the lower part of bednets collected from the field and wrapped in aluminum sheets. Each sample was identified by a code number and kept at 4°C until Bio-assays were performed (less than one month).

#### Mosquito strains

The bio-efficacy of treated nets was assessed on the OC-Lab strain of *A. gambiae *s.s., originated from Yaoundé and laboratory-reared for about 15 years without insecticide selection. The Kisumu susceptible reference strain of *A. gambiae *s.s., originated from Kenya and provided by LIN/IRD Montpellier, was used as a control.

The OC-Lab. strain is known to be strongly resistant to DDT and tolerant to pyrethroids, response to WHO susceptibility test [18] performed in 1997 is given in table 1. We registered 26 per cent mortality rate to 4 per cent DDT, 78–95 per cent mortality rates to 0.25 percent permethrin (former diagnostic concentration), 0.025 per cent deltamethrin (former diagnostic concentration) and 0.2 per cent cyfluthrin. With these diagnostic concentrations, the time of knockdown for 50 per cent mosquitoes during exposure to insecticide-impregnated papers was 2–5 fold increased compared with that of the Kisumu strain. However, mortality rates to 1.0 per cent permethrin, 0.05 per cent deltamethrin (revised current diagnostic concentrations) was higher than 98 per cent, with knockdown time ratio less than 2 fold.

**Table 1:** Kisumu susceptible and OCEAC Laboratory strains of *Anopheles gambiae *s.s. response to WHO susceptibility test.

**Strains**	**Insecticides**	**No**	**TKd_50_(CI)**	**TKd_95_(CI)**	**Tkd_50_R (CI)**	**Mt**	**ST.**
**Kis.**	4% DDT	100	18.8 (17.6–20.0)	28.7 (25.8–33.7)	--	100	S
	1.0% permethrin	99	9.2 (8.6–9.7)	14.3 (13.2–16.0)	--	100	S
	0.25% permethrin	100	12.4 (11.2–13.7)	28.8 (24.8–35.4)	--	94.1	T
	0.05% deltamethrin	89	9.4 (8.4–10.2)	17.2 (15.6–20.0)	--	100	S
	0.025% deltamethrin	100	8.9 (8.1–9.7)	19.7 (17.5–23.0)	--	100	S
	0.2% cyfluthrin	120	8.6 (8.0–9.1)	15.5 (14.1–17.7)	--	100	S
**OC-Lab.**	4% DDT	100	9.9 (86.1–119.6)	268.9 (195.0–465.5)	5.2 (4.0–6.7)	26	R
	1.0% permethrin	101	12.2 (11.5–12.8)	17.5 (16.3–19.5)	1.3 (0.9–1.7)	98.7	S
	0.25% permethrin	125	45.7 (13.6–153.3)	109.2 (3.3–3593.0)	3.7 (0.6–22.9)	78.7	R
	0.05% deltamethrin	100	16.8 (13.2–21.3)	36.2 (23.7–55.5)	1.8 (1.1–2.9)	100	S
	0.025% deltamethrin	125	24.9 (23.6–26.5)	41.2 (37.5–46.9)	2.8 (2.3–3.4)	94.8	T
	0.2% cyfluthrin	108	17.8 (12.7–24.8)	34.1 (32.5–37.3)	2.1 (1.3–2.9)	94.4	T

Kis.: Kisumu strain, OC-Lab.: OCEAC Laboratory strain, No: Number of tested mosquitoes, Tkd_50_: knockdown time in minutes for 50% tested mosquitoes, Tkd_95_: knockdown time in minutes for 95% tested mosquitoes, CI: confidence interval at 95%, Tkd_50 _R: Tkd_50 _OC-Lab strain / Tkd_50 _Kisumu strain, Mt.: Mortality rate 24 h post exposure, ST: status, S: indicates susceptibility, T: suspects resistance to be confirmed (or tolerance), R: suggests resistance.

**Figure 1:**
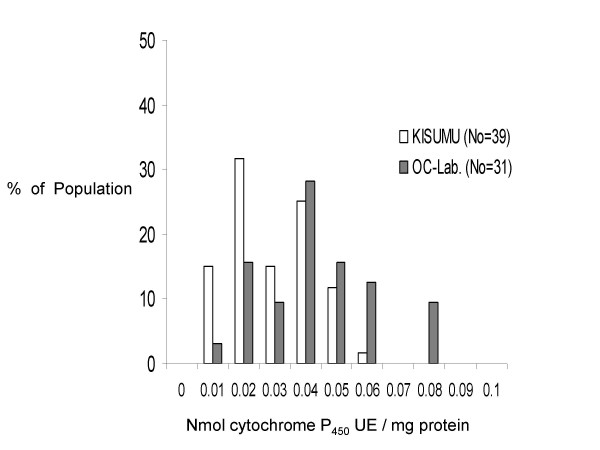
**Oxidase levels in Kisumu and OCEAC laboratory strains of *Anopheles gambiae *s.s. through biochemical assays. **KISUMU: Pattern of cytochrome P_450 _UE/mg protein in individuals of the Kisumu susceptible laboratory strain, OC-Lab: Pattern of cytochrome P_450 _UE/mg protein in individuals of the OCEAC laboratory strain.

Biochemical analysis of esterase, mixed function oxidase and glatathione S-transferase enzyme systems using microtitre plates and spectrophotometer as described by Penilla et al. [19] and Brogdon et al. [20] revealed elevation of mixed function oxidases activity in the OC-Lab strain (Figure 1). For this enzyme system, the activities (mean ± standard deviation) of the OC-Lab and Kisumu strains were 0.049 ± 0.018 and 0.027 ± 0.011 nmol cytochrome unity equivalent/mg protein (rank-sum normal statistic with correction Z = -3.924, *p *< 0.001). Esterase and glutathione S-transferase levels were lower in the OC-Lab strain than in the Kisumu strain. For esterases, two substrates were used, α-naphtyl acetate and paranitrophenyl acetate. With α-naphtyl acetate, the activities of the OC-Lab and Kisumu strains were 0.057 ± 0.011 and 0.117 ± 0.083 μmol α-naphtol produced/min/mg protein (rank-sum normal statistic with correction Z = 6.302, *p *< 0.001).

With paranitrophenyl acetate, the activities of the OC-Lab and Kisumu strains were 0.002 ± 0.008 and 0.053 ± 0.132 μmol *p*-nitrophenol produced/min/mg protein (rank-sum normal statistic with correction Z = 7.028, *p *< 0.001). For glutathione S-transferases, the activities of the OC-Lab and Kisumu strains were 0.013 ± 0.024 and 0.087 ± 0.104 μmol GSH conjugated/min/mg protein (rank-sum normal statistic with correction Z = 3.172, *p *= 0.001).

PCR analysis [21] showed that individuals of the OC-Lab strain belonged to the M molecular form of *A. gambiae *s.s. and those of the Kisumu strain to the S molecular form. Individuals of the OC-Lab. strain which survived to WHO susceptibility test were screened by PCR [22], all of them appeared free of kdr Leu-Phe mutation.

#### Bio-assays

After treatment, ten batches of five unfed females (2–5 days old) of the OC.Lab strain and those of the Kisumu strain were exposed under WHO's plastic cones to netting from newly treated nets for three minutes. Ten other batches of each mosquitoe strain were exposed to netting from untreated nets as control. Mosquitoes were then transferred in white cups and the knockdown rates were recorded at 60 minutes post-exposure. They were then supplied with a 15% glucose solution and held under laboratory conditions, at 80 per cent relative humidity and 27°C (± 2°C) temperature. The mortality rates were recorded after 24 hours.

Bio-assays were also performed on used nets. Between March and September 1998, 100 females of *A. gambiae *s. s. from the OC-Lab strain and 100 specimens from the Kisumu strain were tested each month (from M_0 _to M_6_). For the control, 50 specimens from the OC-Lab and 50 others from the Kisumu strain were exposed to untreated netting.

Each month knockdown and mortality rates of the OC-Lab strain and the Kisumu strain were then compared by means of the Mantel-Haenszel chi-square test.

## Results

### Efficacy of freshly treated nets

Table 2 indicates knockdown and mortality rates in mosquitoes after exposure to nettings from freshly treated and untreated nets.

**Table 2:** Kisumu and OCEAC Laboratory strains of *Anopheles gambiae *s.s. response to permethrin EC freshly treated nets.

**Variables**	**Nets**	**Kisumu strain**		**OC-Lab strain**		**X**^2^	***p***
				
		No	%	No	%		
**Kd rates**	Untreated	50	0	50	0		
	Permethrin EC (500 mg/m^2^)	100	100	100	97	3.04	0.08
**Mt rates**	Untreated	50	0	50	2		
	Permethrin EC (500 mg/m^2^)	100	89	100	68	13.06	<0.001

Kd: Knockdown rates 60 minutes post-exposure, Mt: Mortality rates 24 hours post-exposure, No: number of tested mosquitoes, *p*: Probability at 5%.

#### Knockdown Rates

No knockdown effect was observed in mosquitoes exposed to netting from untreated nets (control), either in the Kisumu strain or in the OC-Lab strain. With netting from permethrin EC freshly treated nets (M_0_), more than 95 per cent of mosquitoes from both strains were knocked down 60 minutes post-exposure. The difference between the two strains was not significant at the five per cent level (*p *= 0.08, df = 1).

#### Mortality rates

With netting from untreated nets, mortality rate in each strain did not exceed 2 per cent. Using netting from permethrin EC freshly treated nets, the mortality rate in the OC-Lab strain did not exceed 70 per cent, while about 90 per cent mosquitoes of the Kisumu strain were killed, difference between the two strains was highly significant (*p *< 0.001, df = 1).

### Efficacy of treated bednets during domestic utilization

#### Knockdown rates

**Figure 2:**
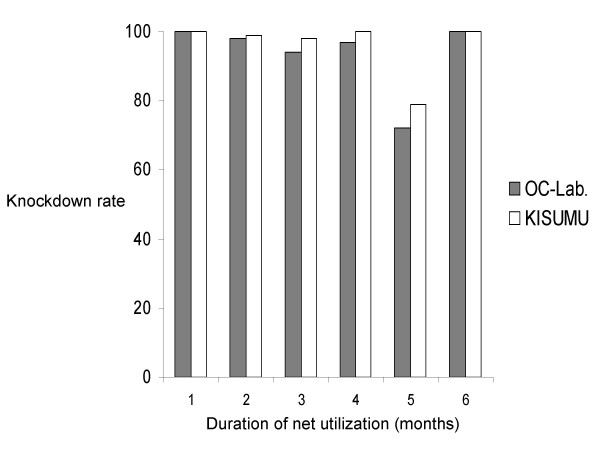
**Knockdown rates in Kisumu and OCEAC laboratory strains of *Anopheles gambiae *s.s. to permethrin EC used nets. **OC-Lab: Permethrin EC treated net knockdown rates on the OCEAC laboratory strain, KISUMU: Permethrin EC treated net knockdown rates on the Kisumu susceptible laboratory strain.

During the whole trial, no knockdown effect was observed in mosquitoes exposed to netting from untreated nets, either in the Kisumu strain or in the OC-Lab strain, while most of the mosquitoes exposed to netting from treated nets were knocked down during the 60 minutes post-exposure. The profile of knockdown rate variations during the six month evaluation is given in Figure 2. No significant difference was observed between the Kisumu strain and the OC-Lab strain (*p *> 0.05, df = 1). For both strains, the knockdown rate was mostly higher than 90 per cent, except in the fifth month during which about 70 per cent were registered.

#### Mortality rates

**Figure 3:**
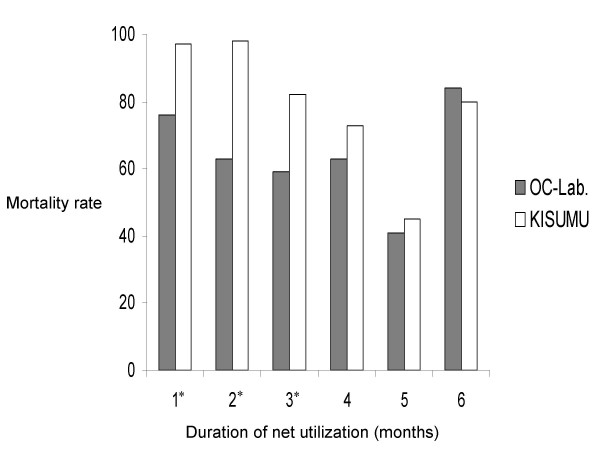
**Mortality rates in Kisumu and OCEAC laboratory strains of *Anopheles gambiae *s.s. to permethrin EC used nets. **OC-Lab: Permethrin EC treated net mortality rates on the OCEAC laboratory strain, KISUMU: Permethrin EC treated net mortality rates on the Kisumu susceptible laboratory strain, * stars indicate months during which the mortality rates were significantly lower in the OCEAC strain than in the kisumu strain.

The mortality rates in the control netting were constantly lower than five per cent for both strains. Conversely, numerous mosquitoes exposed to netting from treated nets were killed during the 24 hours post-exposure. The graph of the mortality rate variations during the six month evaluation is given in Figure 3. During the first five months, the killing effect was higher in the Kisumu strain than in the OC-Lab strain. The decrease of net efficacy on the OC-Lab strain was significant during the first three months (*p *< 0.001, df = 1). From the fourth to the sixth month, there was no longer a significant difference between the two strains (0.13 <*p *< 0.57, df = 1).

## Discussion

DDT resistance in the OC-Lab. strain of *A. gambiae *s.s. which originated from Yaoundé city was not selected in the laboratory. The selective pressure was performed in the field several years prior to the collection of the strain. DDT was used in Yaoundé for residual indoor spraying during the 1950's [23]. Furthermore, Desfontaines *et al*. [24] reported the intensive use of household insecticides containing mixture of compounds such as pyrethrins and pyrethroids (coils, mats, etc ...) in this city for protection against mosquito bites. The first susceptibility tests on the OC-Lab strain were performed in 1997 using WHO's protocol. Samples were tested for 4.00 per cent DDT, 0.25 per cent and 1.00 per cent permethrin, 0.025 per cent and 0.05 per cent deltamethrin, then 0.20 per cent cyfluthrin. Susceptibility tests were carried out with these ranges of pyrethroid dosage because the OC-Lab strain had to be used for the evaluation of cyfluthrin bio-efficacy in phase III of the World Health Organization Pesticide Scheme (WHOPES). This strain was found resistant to 4.00 per cent DDT and 0.25 per cent permethrin, tolerant to 0.025 per cent deltamethrin and 0.20 per cent cyfluthrin, but susceptible to 1.00 per cent permethrin and 0.05% deltamethrin. It was also shown that the *kdr *Leu-Phe mutation was not involved in this case of DDT resistance or pyrethroid tolerance. Bio-assays using WHO cone test with a cyfluthrin EW 50 mg active ingredient per m^2 ^of netting resulted in 35 per cent mortality rate versus 95 per cent rate for the Kisumu strain. The difference in knockdown rates was not significant (95–100 per cent for both strains). It was seen that the strain was not suitable for that trial. In fact, the cyfluthrin bio-efficacy was assessed with the Kisumu susceptible reference strain [25]. Subsequently, biochemical analysis revealed over-production of mixed function oxidases in the OC-Lab strain and the same metabolic-based resistance was reported in wild populations of *A. gambiae *s.l. from cotton and rice fields in northern Cameroon [16], which is a threat for the efficacy of treated nets in this area. It was, therefore, essential to investigate pyrethroid-treated material effectiveness against a metabolic-based resistant malaria vector population. With these rationales, the OC-Lab strain was found suitable for a laboratory trial compared with a susceptible reference strain of *A. gambiae *s. s., such as the Kisumu strain. The study was not carried out with field mosquitoes because the cotton fields are actually located 1,000 km from the laboratory, it would be difficult to collect sufficient field samples for bio-assays.

From current data, the insecticide activity of treated nets on the Kisumu reference strain was clearly demonstrated, despite some breakdowns observed after the third month. The decrease of knockdown and mortality rates at this period may be related to bad conditions of net utilization. Previous reports have underlined the impact of external factors such as dirt and fume on the bio-efficacy of treated nets [26-28]. However, the activity of permethrin in this study was similar to that usually reported in field trials [29,30]. Conversely, nets were less effective against the OC-Lab strain, especially in term of mortality rate. These data are consistent with those previously obtained with cyfluthrin.

The decrease of knockdown rate prior to that of mortality rate is known as one of the major modifications of pyrethroid effects associated with kdr mutation [12,31]. The contrast between knockdown and mortality rates in this trial is relevant to the involvement of metabolic detoxification in insecticide resistance which does not systematically induce the decrease of knockdown effect.

In Cameroon, ITNs were found effective in reducing malaria transmission and morbidity during the early 1990s [32,33] and, until now, they have been strongly advocated by the national malaria control programme. Therefore, the emergence of pyrethroid resistance in the *A. gambiae *complex [34] is of a particular concern for the efficacy of interventions. Generally, insecticide resistance has a major impact in reducing efficacy of IRS programmes. Detoxification through mixed function oxidases was reported to delay the deltamethrin IRS programme against *A. funestus *populations from northern Kwazulu/Natal [10]. By the same token, high activities in glutathione S-tranferases, esterases and mixed function oxidases resulted in the failure of the IRS programme against *A. albimanus *in southern Mexico [19]. ITNs tested in laboratory and experimental huts in West Africa were found partially effective against DDT or pyrethroid resistant populations of *A. gambiae *s.s. with *kdr *gene frequency higher than 70% [35]. Nevertheless, the epidemiological impact at community level was similar to that observed in areas with susceptible vectors (Henry, personal communication). The lessening of pyrethroid exito-repellent and irritancy effects against knockdown resistant mosquitoes allowed their contact with treated nets and resulted in killing many of them. ITNs could, therefore, work positively against pyrethroid-resistant malaria vectors with *kdr *gene. Considering the genetic diversity of pyrethoid resistance mechanisms, the efficacy of ITNs against knockdown resistant populations could not be extrapolated to vector populations with elevated monoxygenases activity.

From the current study, it has been seen that the knockdown rates were not decreased in the permethrin-tolerant strain; likewise pyrethroid properties (excito-repellent and irritancy) may not be impeded against metabolic-based resistance mosquitoes. There is a converging suggestion that the impact of insecticide resistance on the efficacy of ITNs used as personal protection tools might not be limited when resistance is due to high metabolic detoxification. Conversely, the decrease of mortality rates puts forward a potential limited impact of ITNs when used at community level as vector control intervention aiming at mass reduction of vector density.

## Conclusions

Current data call attention to early detection of resistance as one of the key guidelines for insecticide resistance management. Susceptibility tests are the entry point for insecticide resistance studies. However, whether resistance is detected or not, it is necessary to go through biochemical or molecular assays for detection of resistance genes which may not have a great impact on the level of resistance when they stand at very low frequency. In order to preserve efficacy of relevant tools such as ITNs against malaria vectors, it would be easy to control insecticide resistance when the occurrence of the involved gene is at low rather than at high frequency.

Moreover, the impact of insecticide resistance on vector control interventions is a complex phenomenon that depends not only on the resistance itself (mechanisms, gene frequency, etc...), but also includes vector behaviour, environment and insecticide properties such as the excito-repellent effect. Drawing a general conclusion on the efficacy of ITNs in areas with metabolic-based resistant vector populations needs further investigation in experimental huts to study their behaviour and at community level to assess epidemiological impact.

## Authors' contributions

JE participated in the conception of the study, carried out field and laboratory procedures, analysed and interpreted data, drafted and revised the manuscript.

FC carried out biochemical assays, participated in data analysis and interpretation. PG participated in the design of the study and revising it critically. LM conceived the study, participated in its design, coordinated, revised and gave final approval of the version to be published.
